# Novel Roles for Chloride Channels, Exchangers, and Regulators in Chronic Inflammatory Airway Diseases

**DOI:** 10.1155/2015/497387

**Published:** 2015-11-03

**Authors:** Monica Sala-Rabanal, Zeynep Yurtsever, Kayla N. Berry, Tom J. Brett

**Affiliations:** ^1^Department of Cell Biology and Physiology, Washington University School of Medicine, St. Louis, MO 63110, USA; ^2^Center for the Investigation of Membrane Excitability Diseases, Washington University School of Medicine, St. Louis, MO 63110, USA; ^3^Biochemistry Program, Washington University School of Medicine, St. Louis, MO 63110, USA; ^4^Department of Internal Medicine, Washington University School of Medicine, St. Louis, MO 63110, USA; ^5^Drug Discovery Program in Pulmonary and Critical Care Medicine, Washington University School of Medicine, St. Louis, MO 63110, USA; ^6^Medical Scientist Training Program, Washington University School of Medicine, St. Louis, MO 63110, USA; ^7^Department of Biochemistry and Molecular Biophysics, Washington University School of Medicine, St. Louis, MO 63110, USA

## Abstract

Chloride transport proteins play critical roles in inflammatory airway diseases, contributing to the detrimental aspects of mucus overproduction, mucus secretion, and airway constriction. However, they also play crucial roles in contributing to the innate immune properties of mucus and mucociliary clearance. In this review, we focus on the emerging novel roles for a chloride channel regulator (CLCA1), a calcium-activated chloride channel (TMEM16A), and two chloride exchangers (SLC26A4/pendrin and SLC26A9) in chronic inflammatory airway diseases.

## 1. Introduction

The chronic inflammatory airway disease, asthma, and chronic obstructive pulmonary disease (COPD) are significant causes of morbidity and mortality in children and adults. Asthma affects over 300 million people worldwide, and the prevalence is increasing among all demographics. COPD is currently the third leading cause of death in USA. These diseases are hallmarked by a Th2-mediated inflammatory response which drives the three pathologies that contribute to airway obstruction in these diseases: chronic inflammation; airway muscle constriction due to airway hyperreactivity (AHR); and mucus overproduction due to mucous cell metaplasia (MCM). A central feature of these diseases is production of the inflammatory cytokines IL-4 and IL-13, which drive MCM and contribute to AHR.

The inflammatory signaling upregulates the expression of hundreds of proteins in the airway epithelia. A number of these proteins have roles in anion transport across membranes, including chloride channels, channel regulators, and transporters. The identity, function, and elucidated mechanism of action of these proteins have lagged behind their cation channel counterparts. However, recent advances in several technologies, including high throughput screening, have made it possible to consider the development of specific inhibitors and activators for these classes of proteins [1]. The development of such therapeutics, however, requires an intimate knowledge of the roles these proteins play in airway homeostasis and mucociliary clearance. Anion channels play very crucial roles in mucus function. Mucus is composed of 97% water and 3% solids, with the main solid component being the mucin proteins [2]. Mucin proteins are secreted in a dehydrated form and require anion channel activity to instill chloride and bicarbonate ions that ensure proper salination, hydration, and pH of the mucus gel layer. Proper control of this is crucial as is exemplified by the disease cystic fibrosis (CF), which is caused by loss of function mutations to the chloride channel cystic fibrosis transmembrane conductance regulator (CFTR) that produces thick, sticky mucus deficient in mucociliary clearance or innate antimicrobial properties [3].

Here we discuss what is currently known about the function of four exciting, new, and emerging proteins affecting anion channel activity in inflammatory airway epithelia: a chloride channel regulator (CLCA1), a calcium-activated chloride channel (TMEM16A), and two chloride exchangers (SLC26A4/pendrin and SLC26A9). In particular, we focus on recently uncovered contributions to airway diseases and mucus function, in order to answer whether they can be targeted by inhibitors or activators and whether they should be.

## 2. The CLCA Family of Chloride Channel Regulators

The CLCA family of proteins was originally misidentified as calcium-activated chloride channels and has long been associated with chronic inflammatory airway diseases. Their evolving functional identity and the possible role they play in these diseases have only recently been elucidated.

### 2.1. CLCAs: Association with Chronic Inflammatory Airway Diseases

**Figure 1:**
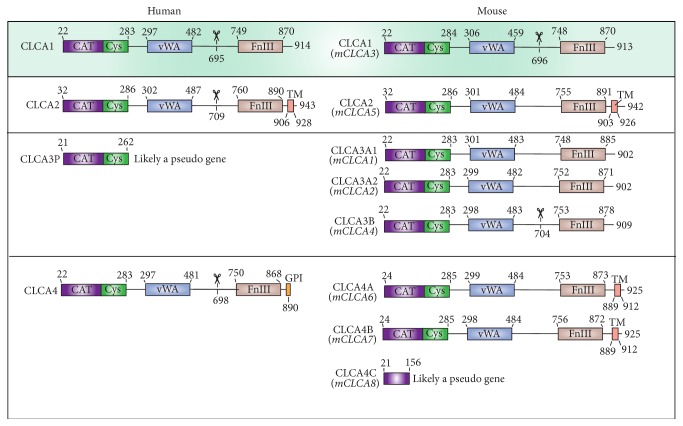
Domain architecture schematic for human and mouse CLCA proteins. Each row contains the corresponding human and mouse homologs. Mouse CLCA proteins are labeled according to the recently updated naming commissioned by the Mouse Gene Nomenclature Committee (MGNC) in order to align the numbering established by the Human Gene Nomenclature Committee and the Rat Genome Database. The previously used names for the mouse proteins are shown below the current names and are in italics. Scissors denote the experimentally determined location of proteolytic cleavage sites [18]. Human CLCA3 and mouse CLCA4C are likely pseudogenes because they contain premature stop codons. Labels denote the following domains: CAT: matrix-metalloprotease-like catalytic domain; CYS: matrix-metalloprotease-like cysteine rich domain; vWA: von Willebrand factor type A domain; FnIII: fibronectin type III domain; TM: transmembrane domain; GPI: glycosylphosphatidylinositol anchor.

Asthmatic inflammation results from a Th2-mediated mechanism, where the cytokines IL-4 and IL-13 bind their receptors and activate the transcription factor STAT6 to drive inflammation and mucus overproduction in the airways [4, 5]. In mouse models of both allergic and respiratory virus induced-asthma, CLCA1 (previously known as mCLCA3 or gob-5) expression has been solidly linked to IL-13 driven MCM [6, 7] and controversially linked to AHR [6], both hallmarks of asthma and COPD. Similar results were observed* in vitro* with the human pulmonary mucoepidermoid cell line NCI-H292, in which expression of the protein significantly increased mucin gene MUC5AC expression and subsequent mucus production [8, 9], implying that CLCA1 can drive MCM. Studies using* Clca1* ^−/−^ mice, however, have failed to show reduced response to IL-13 stimulation, as these mice showed the same phenotype as wild-type (WT) mice [7, 10]. Other members of the family, particularly CLCA2 and potentially CLCA4A and CLCA4B, have also been observed to be upregulated and to induce and colocalize with the inflammatory mucin protein, MUC5AC [7]. These results indicate a possible functional redundancy between members of the mouse CLCA family, which is unlikely to translate to human biology, as there are only three human CLCA proteins, compared to the seven mouse CLCA proteins [11] (Figure 1).

**Figure 2:**
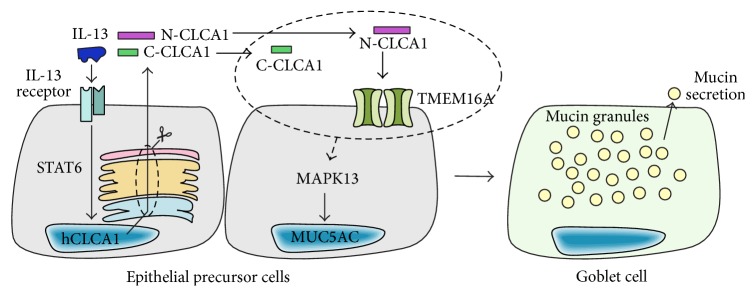
Schematic of CLCA1-driven MCM in human airways based on the current literature. IL-13 induces* CLCA1* gene expression through activated STAT6. CLCA1 protein is expressed, is secreted, and undergoes proteolytic self-cleavage to yield two fragments (N-CLCA1: N-terminal fragment; C-CLCA1: C-terminal fragment). N-CLCA1 engages and activates the CaCC TMEM16A. Downstream, a signaling pathway is activated through MAPK13 which leads to induction of the inflammatory mucin* MUC5AC*, followed by goblet cell differentiation and subsequent MCM. It is currently unknown whether or how the steps highlighted in the dashed ellipse (CLCA1 cleavage and activation of TMEM16A) contribute to the activation of MAPK13.

Of the three human CLCA proteins, CLCA1 has been identified as a potential biomarker of inflammation and MCM in the airways [12] and suggested as a potential drug target for treatment of asthma and COPD. Similar to mouse CLCA1, overexpression of human CLCA1 in NCI-H292 cells increases MUC5AC expression and mucus levels [6, 8, 9]. Its expression is upregulated in primary cell models of IL-13 driven MCM and siRNA-mediated knockdown of CLCA1 prevents IL-13-driven mucus overproduction [9]. These experimental observations suggest a central role for CLCA1 function in IL-13-mediated MCM (Figure 2). Additionally, it is highly overexpressed in the airway epithelia of asthmatic patients [8] and can be found in the bronchoalveolar lavage fluid (BALF) at high levels [13]. In contrast, other members of the human CLCA family are not upregulated in response to IL-13 [8, 9], suggesting that CLCA1 is the sole family member with an essential role in MCM in human airways.

### 2.2. The Conceptual Evolution of CLCA Proteins from “Channels” into “Channel Regulators”

The family of CLCA proteins were first cloned from bovine and murine samples, where overexpression of these proteins increased Ca^2+^-sensitive Cl^−^ conductance, which led to their initial misannotation as Ca^2+^-activated Cl^−^ channels (CaCCs) [14–16]. In addition, observations that nonspecific chloride channel blockers, such as niflumic acid and DIDS, seemed to reduce both currents and the mucus production [17] erroneously supported this hypothesis. However, modern bioinformatics algorithms and experimental approaches have definitively demonstrated that CLCA proteins are soluble, secreted proteins that do not constitute ion channels themselves [13, 18], and subsequent studies demonstrated that CLCA proteins activate currents through an endogenous CaCC [18–20]. As a consequence, the CLCA nomenclature has been updated and the family is now recognized as Ca^2+^-activated Cl^−^ channel regulator proteins. All members of this family (with the exception of the likely pseudogenes human CLCA3 and mouse CLCA4C) are synthesized as full-length proteins and proteolytically cleaved into two fragments. It has been demonstrated that, for some of the family members, the cleavage is carried out by a zinc-dependent matrix metalloprotease-like (MMP-like) domain located in the N-terminus of the protein [18, 21] (Figure 1). However, sequence analysis has revealed that all CLCA family members contain the required MMP active site motif [18]; thus self-cleavage is likely a conserved feature of all CLCAs. Self-cleavage is required to unmask the N-terminal fragment of CLCA1, which then interacts with the CaCC [18] (Figure 2). The molecular identity of this previously unknown CaCC has been shown to be TMEM16A and the direct interaction between the channel and the N-terminal fragment stabilizes and increases the cell surface expression of the channel, thereby increasing currents [20].

### 2.3. CLCA1 as a Potential Regulator of Cytokine Expression

Upstream of CLCA1 expression, the involvement of Th2-cytokines IL-4 and IL-13 has been shown in cellular and animal models as discussed above. The relationship between CLCA1 and downstream cytokine signaling, however, is still under investigation. The few articles that do exist report contradictory observations regarding the role of CLCA1 as a signaling molecule for cytokine expression. Challenging* Clca1* ^−/−^ mice with lipopolysaccharide (LPS), Long et al. observed increased KC (keratinocyte-chemoattractant) levels in BALF, but no change in MIP-2 (macrophage inflammatory protein 2) or IL-17 levels in the knockout mice compared to WT mice [22]. In contrast, Dietert et al. observed significantly decreased levels of IL-17 and CXCL-1 in BALF from the* Clca1* ^−/−^ mice infected with* Staphylococcus aureus* [23]. Using a cellular model of inflammation, Ching et al. showed that CLCA1-conditioned media increased proinflammatory cytokine (IL-6, IL-8, IL-1*β*, and TNF *α*) mRNA levels in monocyte cell line U-937 and primary porcine alveolar macrophages. Immunopurified CLCA1 protein only increased IL-8 and IL-1*β* levels significantly [24]. If such a regulatory mechanism exists for cytokine expression, modulation of CLCA1 function with small molecules to treat mucus cell metaplasia might also alter the inflammatory response in the airways.

## 3. TMEM16: The First Family of CaCCs

While CaCC conductance was a long-observed phenomenon in the airways and could be separated from CFTR currents, the molecular identity of the channels responsible for these currents remained elusive until the late 2000s. The TMEM16/Anoctamin family was identified in 2008 as the first bona fide CaCCs [25–27]. However, based on their electrical and pharmacological characterization, only two of the ten family members, TMEM16A and TMEM16B, displayed properties previously observed for CaCCs in the airways [28, 29], whereas most of the other members function as lipid scramblases. Of these two, TMEM16A expression has been verified in airway epithelium and airway smooth muscle cells [30].

### 3.1. TMEM16A Is Linked to Chronic Inflammatory Airway Diseases

**Figure 3:**
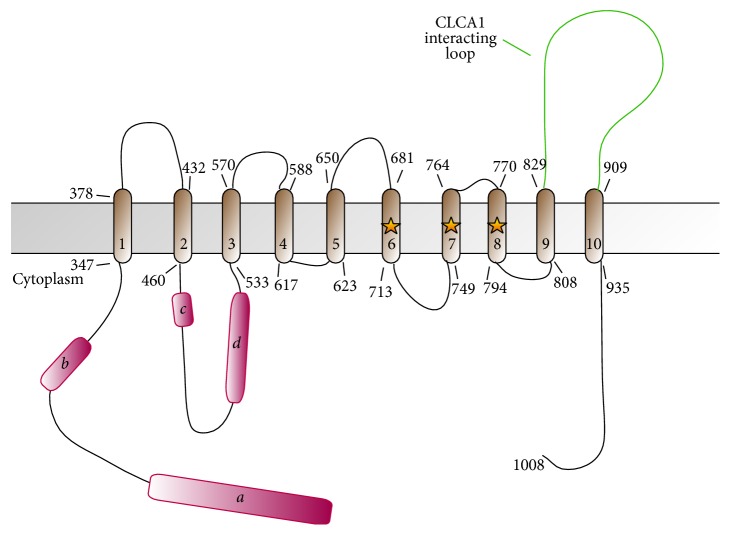
Domain architecture schematic for human TMEM16A. Topology shown is predicted from structure-based alignment to the crystal structure of the fungal* Nectria haematococca* TMEM16 [31]. Alternative splicing segments *a*, *b*, *c*, and *d* are shown in magenta. The location of residues of a crystallographically determined Ca^2+^ binding site is highlighted with stars. The extracellular loop mediating interaction with CLCA1 is highlighted in green.

The predicted topology for TMEM16 family members is based on the recent landmark crystal structure of the fungal* Nectria haematococca* TMEM16 (nhTMEM16) which has 10 transmembrane domains instead of the previously predicted 8 (Figure 3) [31]. The purified and reconstituted protein, which was shown to be a homodimer [32], constitutes a channel on its own and does not require other proteins to be active [33]. While the Ca^2+^ sensitivity of the channel is well documented [31, 34, 35] and the protein directly binds Ca^2+^, the possible involvement and mechanism of interaction with calmodulin as a calcium sensor and binding partner are still controversial [35–37].

Much like CLCA1, expression of TMEM16A is upregulated by IL-4/IL-13 stimulation [38–41]. Upon upregulation, TMEM16A colocalizes to the apical plasma membrane of goblet cells along with the mucin MUC5AC [41–43]. It is expressed in airway smooth muscle cells and has been shown to play a role in AHR [42]. Additionally, inhibitors of TMEM16A have been shown to block mucus secretion [42, 44] whereas small molecule activators increase secretion [45].

### 3.2. The Potential for Targeting CLCA1 and TMEM16A in Chronic Inflammatory Airway Diseases

CLCA1 is a promising therapeutic target for asthma and COPD, as it is the only member of its family to be upregulated in models of IL-13 mediated mucus overproduction [9], is a secreted protein [18], is expressed in goblet cells [7, 9, 16], and associates with mucin granules [46, 47]. Overexpression of CLCA1 in airway epithelium induces MUC5AC expression via a signaling cascade involving the kinase MAPK13, and siRNA-knockdown of CLCA1 blocks mucus production in these models [9], implying a critical role for CLCA1 function in inflammatory mucus production. Consistent with these observations, DNA vaccines [48] and antibodies targeting mouse CLCA1 [49] have displayed some effectiveness in reducing airway inflammation and MUC5AC levels in mouse asthma models. Similarly, as mentioned above, TMEM16A inhibitors block ATP-dependent mucus secretion, suggesting a central role for this pair in inflammatory mucus overproduction. However, it should be noted that these small molecules have relatively low potency and questionable selectivity and these findings need to be supported by additional experiments to determine the role TMEM16A plays in mucus secretion [50]. An important question that currently remains unanswered is what role this pair of molecules plays in mucociliary clearance. It is well known that anion channel activity is required for secreted mucins (MUC5AC and MUC5B) to function properly in a mucosal immunity and mucociliary clearance capacity [51]. Mucin proteins are secreted in dense, dehydrated granules and require anion channel passage of chloride and bicarbonate ions to ensure proper hydration, salination, and pH control [2]. Thus, any therapy targeting anion channel activity in airway diseases should proceed with caution to avoid any potential detrimental impacts to mucociliary clearance and innate mucosal immunological properties. Along these lines, exploiting the mechanism CLCA1-mediated regulation of TMEM16A action might be a possible therapeutic route for CF, utilizing a potential compensatory channel to make up for loss of CFTR activity [52]. Further along these lines, TMEM16A has been shown to carry not only chloride ions but also bicarbonate ions [36]; thus activation of TMEM16A in the setting of CF could be beneficial to adjust mucus hydration and pH.

## 4. SLC26: An Ancient Family with Unexpected New Roles

Over the last decade the anion exchanger pendrin (PDS,* SLC26A4*), once thought to be limited mainly to the inner ear, kidney, and thyroid, has been found to be upregulated by inflammatory cytokines in the bronchial epithelium, where it contributes to the pathogenesis of inflammatory airway diseases [53–55] and also to the host response to bacterial infections [56, 57]. Another member of the family, SLC26A9, is prominently expressed in the airway epithelia, where it interacts with CFTR to modulate mucus production [58]. The discovery of these crucial roles in lung physiology and pathophysiology makes these anion transporters intriguing new biomarkers for airway disease and promising novel pharmacological targets.

### 4.1. Pendrin: An Anion Exchanger with Critical Roles in Ear, Kidney, and Lung Physiology

**Figure 4:**
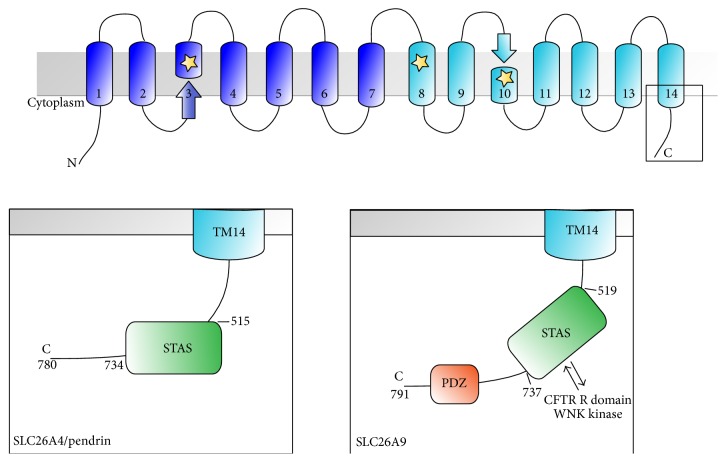
Domain architecture schematic for human SLC26 family proteins discussed here, SLC26A4 (pendrin) and SLC26A9, based on the crystal structure of SLC26Dg (PDB ID 5DA0). Upper inset shows general schematic while the lower insets show details of the C-terminal cytoplasmic region for each protein. Labels denote the following domains: STAS: sulfate transporter and antisigma factor antagonist domain; PDZ: PSD95-Dlg1-Zo-1 domain. Locations of SLC26A9 interaction with CFTR R domains and WNK kinases are denoted. The location of residues of a crystallographically determined ligand binding site is highlighted with stars.

Pendrin (PDS,* SLC26A4*) is a member of the SLC26 family of multifunctional anion transporters and channels [59, 60]. The eleven mammalian* SLC26* genes encode proteins with cytoplasmic N-termini and C-termini flanking a transmembrane core of unknown structure, predicted to span the lipid bilayer 10 to 14 times (Figure 4). Mutagenesis, homology modeling, and molecular dynamics simulation data are consistent with the hypothesis that the SLC26 transmembrane fold consists of two nesting, inverted repeats of 5–7 helices, resembling that of the CLC Cl^−^/H^+^ antiporter channel proteins [61, 62] and the recently solved three-dimensional structure of SLC26Dg, a bacterial H^+^-coupled fumarate symporter, has clarified this [63]. It has been suggested that SLC26 proteins organize in functional homodimers or homotetramers [64], though each subunit is thought to constitute an independent translocation pathway. The C-terminal cytoplasmic region of all SLC26 proteins includes a sulfate transporter and antisigma factor antagonist (STAS) domain (Figure 4), which has been implicated in nucleotide binding and hydrolysis [65]. SLC26A4, or pendrin, functions as an electroneutral exchanger of Cl^−^, HCO_3_^−^, I^−^, NO_3_^−^, formate, SCN^−^, and other monovalent anions. It is expressed in cochlear epithelial cells of the spiral prominence, in root cells, in spindle cells of the stria vascularis, in epithelial cells of the endolymphatic sac, and in epithelial cells surrounding the hair cells of the saccule, utricle, and ampulla [66]. Additionally, pendrin is expressed in the apical membrane of thyrocytes [67, 68], renal collecting duct Type B intercalated cells [69], salivary gland cells [70], and airway epithelia [53].

**Figure 5:**
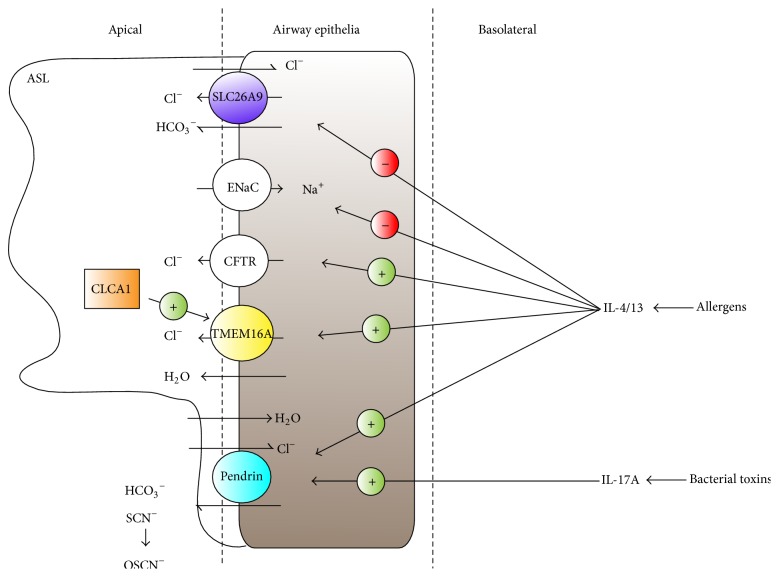
Roles of ion channels in the airway epithelium. In response to allergens and other asthma and COPD exacerbating factors, Th2-cytokines IL-4 and IL-13 induce a water secretory phenotype by stimulating Cl^−^ secretion via CFTR and Ca^2+^-activated Cl^−^ channels such as TMEM16A and by decreasing Na^+^ and Cl^−^ reabsorption via ENaC and SLC26A9, respectively, which leads to the thickening of the airway surface liquid (ASL). TMEM16A activity can be increased by secreted CLCA1 protein. In concert, signaling through IL-4/IL-13 increases the functional expression of pendrin, which results in reabsorption of water and thinning of the ASL. Pendrin can also increase the secretion of thiocyanate (SCN^−^), a substrate for the production of the antimicrobial agent hypothiocyanite (OSCN^−^) by the lactoperoxidase system. Toxins from* B. pertussis* and other bacteria trigger an IL-17A-mediated inflammatory host response in the lung epithelium, which is characterized by a significant upregulation of pendrin activity.

Pendrin function is important in several settings. In the inner ear, pendrin helps maintain Cl^−^ and HCO_3_^−^ homeostasis, which is crucial for normal hearing and for the development of bony structures such as the cochlea and the vestibular aqueduct [71]. In the thyroid gland, pendrin contributes I^−^ to the follicle for thyroxine biosynthesis [72], and in the cortical collecting duct the transporter is implicated in Cl^−^ reabsorption through functional coupling with the epithelial Na^+^ channel ENaC and the Na^+^-dependent Cl^−^/HCO_3_^−^ exchanger NDCBE/SLC4A8 [73]. Most interestingly, it has been shown that in the bronchial epithelium pendrin mediates an increase in Cl^−^/SCN^−^ exchange in response to IL-4 stimulation [74] to provide SCN^−^ substrate to lactoperoxidase for the synthesis of hypothiocyanite (OSCN^−^), a molecule with antimicrobial properties [75], and this underscores the emerging role of pendrin in innate airway defense mechanisms (Figure 5).

### 4.2. Pendrin and the Pathogenesis of Inflammatory Lung Disease: Too Much of a Good Thing?

Pendrin was first identified by positional cloning as the disease gene for Pendred syndrome (OMIM number 247600), an autosomal recessive condition characterized by deafness with enlargement of the vestibular aqueduct, complex abnormalities in cochlear structure, and variably penetrant euthyroid goiter [76–78]. Pendrin is also implicated in DFNB4, an autosomal recessive form of nonsyndromic deafness [79].* SLC26A4* mutations that are clinically associated with Pendred syndrome cause complete loss of transport function when studied in heterologous expression systems, mostly due to retention in various intracellular compartments, whereas those exclusively associated with DFNB4 have residual transport activity [80]. In recent years, pendrin* gain* of function, mainly due to increased surface expression, has been linked to respiratory diseases including bronchial asthma, COPD, and rhinovirus infection, rhinitis, and chronic rhinosinusitis [54, 55, 81–86].

The association between pendrin and inflammatory airway disease was first proposed in 2005, when it was observed that pendrin expression was upregulated in three different murine asthma models, including transgenic overexpression of IL-13 in lung [83]. Later, it was reported that induction of asthma or COPD in mice by inhalation of ovalbumin or elastase, respectively, resulted in increased pendrin expression; direct overexpression of pendrin in the lung led to increased mucus production and secretion and neutrophilic infiltration [86]. In subsequent works, the link between inflammatory cytokines, in particular IL-4 and IL-13, and pendrin overexpression has been cemented [81, 85, 87], and in a recent study* SLC26A4* was identified as the most upregulated gene in human asthmatic bronchi [88]. A major downstream effect of IL-4 and IL-13 signaling is the activation of the signal transducer and activator of transcription 6 (STAT6). Following ligand-receptor binding, associated Janus kinases (JAKs) activate the receptor, allowing STAT6 to then be recruited and activated by phosphorylation. Once phosphorylated, STAT6 homodimerizes and translocates to the nucleus where it regulates the transcription of target genes via binding to N4 interferon-*γ* activated sequences (N_4_ GAS) in the promoter region [89]. The pendrin promoter contains at least one N_4_ GAS motif, and STAT6 has been shown to bind this sequence* in vitro*, thus suggesting that increases in pendrin promoter activity via STAT6 represent at least one mechanism by which IL-4 and IL-13 increase pendrin activity [87]. Cytokines other than IL-4 and IL-13 may be responsible for increases in pendrin expression; IL-1*β*, a macrophage-secreted cytokine involved in the immunopathogenesis of asthma and COPD, has also been shown to increase pendrin levels in rodent and human bronchial epithelial cells [74, 90].

Signaling through IL-4/IL-13 mediates airway hyperresponsiveness, eosinophilic inflammation, mucus cell metaplasia and mucus overproduction, subepithelial fibrosis, and increased viscosity of the airway surface liquid (ASL), all of which are common to bronchial asthma and COPD [91]. Pendrin may play a major role in the pathogenesis of asthma or COPD by regulating some of these responses, in particular ASL thickness and mucus production (Figure 5). In lung epithelial cells, reabsorptive Na^+^ transport through ENaC is suppressed whereas secretory Cl^−^ transport through CFTR and CaCCs is stimulated, which collectively results in a net secretory phenotype whereby water osmotically flows into the lumen and ASL viscosity decreases. On the other hand, pendrin imports Cl^−^ in exchange for other anions, and thus an IL-4/IL-13-mediated increase in pendrin activity may shift the equilibrium towards a reabsorptive phenotype, resulting in the osmotic flow of water into the interstitium and the thinning of the ASL [85]. In asthmatic mice, mucus overproduction is accompanied by an increased pendrin expression at the apical surface of bronchial epithelial cells, and in pendrin overexpression cell models, production of MUC5AC, a major mucus protein in asthma and COPD patients, is increased [86]. In mice, pendrin overexpression is also accompanied by neutrophil-dominant inflammation, suggesting that, in this system, mucus production may be induced not only by a direct effect of pendrin on airway epithelial cells, but also by an indirect effect of pendrin by recruiting inflammatory neutrophils [86].

In bronchial epithelial cells, IL-4/IL-13 signaling upregulates the expression of CLCA1 [9], CFTR [92], and pendrin [87], whereas it downregulates the expression of the *β* and *γ* subunits of ENaC. Because upregulation of certain proteins, such as pendrin, might aggravate asthma or COPD symptoms, whereas the downregulation of ENaC and the downregulation of CLCA1 might be protective (Figure 5), it is unsurprising that pharmacological strategies aimed at the blocking of the IL-4/IL-13 pathway are not as successful as they were anticipated to be [93]. Selective inhibition of pendrin could be an intriguing new strategy for asthma/COPD therapy, but as noted above, pendrin contributes to the secretion of SCN^−^, a substrate of lactoperoxidase for the production of the protective, antimicrobial OSCN^−^ [74], and this should be taken into account when exploring novel treatment avenues.

### 4.3. The Next Frontier: Pendrin and Infectious Lung Disease

Most recently, pendrin has been implicated in the IL-17A-dependent host inflammatory response to bacterial airway infections (Figure 5) [56, 57]. IL-17A is critical for the immune response of the lung to infectious bacteria, for example,* Haemophilus influenzae*,* Staphylococcus aureus*,* Klebsiella pneumoniae,* and* Bordetella pertussis* [94]. The latter is the etiologic agent of whooping cough, or pertussis disease, which is a resurgent condition of great clinical concern as it can progress to pulmonary inflammation and death in infants and for which there is no effective treatment [95]. Pertussis toxin (PT), the virulence factor of* B. pertussis*, undermines the host immune system by inhibiting macrophage and neutrophil responses, suppressing the production of antibodies against the bacteria, and inducing proinflammatory cytokines, in particular IL-17A [96]. One of the most highly upregulated genes in association with PT activity is* SLC26A4* [97, 98], and pendrin is upregulated in human bronchial epithelial cells exposed to IL-17A [56]. In the lungs of* B. pertussis*-infected mice there is an increase in pendrin levels that is concomitant with an increase in IL-17A but not in IL-4/IL-13 levels, and pendrin upregulation is significantly hampered in* Il-17a*-null mice [57]. Other host factors may be involved in PT-dependent upregulation of pendrin, such as IL-1*β* and IFN-*γ*, as both are upregulated during* B. pertussis* infection and have been linked with pendrin upregulation [85, 98, 99]. Taken together, these recent advances suggest that the upregulation of pendrin, with its associated inflammatory pathology, is a major mechanism of virulence for the pertussis toxin and position pendrin as a potential novel therapeutic target for the treatment of whooping cough.

### 4.4. SLC26A9: A Novel CFTR Regulator

SLC26A9 is another member of the SLC26 family of anion exchangers and channels. It is robustly expressed in apical airway epithelia [100] and gastric parietal cells [101] and to a lesser extent in the kidney, brain, and reproductive tracts [59, 102–104]. The function of SLC26A9 is still unclear: it has been described as a Cl^−^ channel with a small degree of bicarbonate transport [104], a Cl^−^ channel or a Cl^−^/HCO_3_^−^ exchanger [105, 106]. One group reported increased Cl^−^ conductance particularly in high bicarbonate conditions [107], and others have found that SLC26A9 activity is coupled to Na^+^ transport [58, 103, 108]. Like other members of the family, SLC26A9 is predicted to span the membrane 14 times, and it contains a STAS domain followed by a PSD95-Dlg1-Zo-1 (PDZ) domain in the C-terminal cytoplasmic region (Figure 4) [59, 109]. Two groups propose that SLC26 proteins, including SLC26A9, interact with CFTR initially via their PDZ domains [110, 111]. This is followed by a stronger interaction between the CFTR R domain and the SLC26 STAS domain, which is enhanced by PKA-dependent phosphorylation of the R domain [111–113]. The interaction between SLC26A9 and CFTR has been described in multiple studies. However, whether the interaction is stimulatory or inhibitory is still controversial and may be cell type-dependent [108, 114]. Not much is known about the regulation of SLC26A9, but WNK kinases, also known to regulate other transporters and channels involved in osmoregulation, have been shown to inhibit SLC26A9 activity via interaction with the STAS domain [109]. Though the influence of SLC26A9 on CFTR has been reported, the reciprocal interaction is less clear. Multiple groups have described CFTR regulation of SLC26A9 activity and expression, but the results are not consistent [58, 114–118]. Evidence of CFTR and SLC26A9 coexpression has been found in the lung, trachea, stomach, and sweat gland [119].

Due to its high expression in the lung, numerous studies have investigated the role of SLC26A9 in lung disease. Anagnostopoulou and colleagues [120] first reported that SLC26A9 activity is responsible for increased constitutive Cl^−^ current under Th2 inflammatory conditions, but not in normal physiology. The authors also found that SLC26A9 prevents airway mucous obstruction after stimulation with IL-13. These changes were due to increased SLC26A9 activity, which may be due to changes in regulation by WNK kinases [104]. Unlike CFTR and TMEM16A, SLC26A9 is downregulated in patients with allergic asthma (Figure 4). Further investigation revealed that a SNP in the 3′ UTR of the* SLC26A9* gene likely reduced expression levels in these patients, possibly through enhanced binding of hsa-miR-632 [120].

SLC26A9 has also been implicated in the pathogenesis of bronchiectasis, the widening of airways frequently due to mucous obstruction, a condition often seen in patients with cystic fibrosis. A recent report [119] identified two patients with diffuse idiopathic bronchiectasis who also had mutations in the* SLC26A9* gene. One patient presented with a mutation in a transmembrane domain of SLC26A9 (V486I); the patient's brother was asymptomatic, though he had the same mutation. The second patient presented with a mutation in the STAS domain of SLC26A9 (R575W) in addition to the Fdel508 mutation in CFTR. Coexpression of both mutants in* Xenopus* oocytes provided evidence of a decreased interaction between the SLC26A9 STAS domain and the R domain of CFTR. It is thought that wild-type SLC26A9, in conjunction with CFTR loss, may enhance ion conductance and fluid secretion [121]. Thus, loss of SLC26A9, in the setting of CFTR loss, may result in reduced airway surface liquid hydration, mucous blockage, and consequent bronchiectasis. The authors of the study further narrow the CFTR-SLC26A9 interaction region to a peptide within the STAS domain but do not confirm that the R575W mutation in this peptide disrupts the interaction and activation [119]. In contrast, a second group did not report changes in Cl^−^ transport with the R575W mutation [122]. As the second patient's daughter only carried the Fdel508 mutation and was asymptomatic, the authors speculate that one mutation in CFTR is not sufficient to produce the CF phenotype [119]. However, mutations in modifier genes, such as SLC26A9, may contribute to CF in those heterozygous for CFTR mutations. Thus, SLC26A9 may influence phenotypic expression of heterozygous mutations in ion channels (CFTR, ENaC, and others) involved in airway surface liquid hydration [119].

Further supporting the modifier gene hypothesis, some SLC26A9 mutations have been shown to increase the risk of meconium ileus in patients with CF [123], as well as CF-related diabetes onset [124] and pancreatic disease severity [108, 125]. In the case of CFTR and SLC26A9 double mutations, the exact mechanism causing the phenotype must be further investigated as Cl^−^ transport could be due to altered CFTR and/or SLC26A9 function or to impaired regulation of CFTR by SLC26A9 or vice versa [119, 124]. Understanding the various mechanisms of SLC26A9 mutations will be important towards developing therapies that can improve lung diseases such as asthma, CF, and bronchiectasis.

## 5. Conclusions

Chloride transport proteins play crucial roles in airway health and disease. On one hand, they contribute to proper mucus function by controlling mucus hydration and pH via controlling chloride and bicarbonate ion transport. On the other hand, they may play a direct role in mucus synthesis, secretion, and AHR. Recent animal model studies have emphasized the crucial role that mucus and mucin proteins play in innate mucosal immunology. Deletion of MUC5B (the main secreted mucin protein produced in the airway under homeostatic conditions) results in impaired mucociliary clearance and increased microbial infection [126]. Knockout of CFTR in pig results in airway mucus that is more acidic and deficient in antimicrobial activity due to loss of defensin function at low pH [3, 127] and is also deficient in mucociliary clearance as it remains tethered to secreting cells [128]. This could be due to improper proteolytic processing of mucin proteins, since loss of CFTR function in the intestine impairs *β*-meprin processing and release of secreted mucins in that setting [129]. Thus a complete understanding of how these channels contribute to mucus synthesis, secretion, function, and mucociliary clearance is required to understand the impact of modulating their activity.
